# Should a Dorsal or Anal Scale Be Used for Microcharacteristics Analysis in *Coreius guichenoti*?

**DOI:** 10.3390/ani16121757

**Published:** 2026-06-06

**Authors:** Fengling Zhang, Yang Chen, Weijie Cui, Li Xu, Jianguang Qin, Tao He

**Affiliations:** 1 College of Fisheries, Southwest University, Chongqing 400175, China; nefelibata6@email.swu.edu.cn (F.Z.); cy6666666@email.swu.edu.cn (Y.C.); cuiweijie@email.swu.edu.cn (W.C.);; 2 College of Science and Engineering, Flinders University, Adelaide, SA 5001, Australia; jian.qin@flinders.edu.au; 3 Key Laboratory of Freshwater Fish Reproduction and Development (Ministry of Education), Key Laboratory of Aquatic Science of Chongqing, Chongqing 400715, China

**Keywords:** scale, microcharacteristics, Fourier coefficient, principal component analysis

## Abstract

Fish scales are increasingly used as non-lethal alternatives to otoliths for reconstructing fish life histories. However, spatial variations in scale characteristics across the fish body remain poorly understood. This study compared the morphology and microchemistry (Mg, Ca, Sr, Mn, and Ba) of anal and dorsal scales in *Coreius guichenoti* using elliptic Fourier analysis (EFA) and Inductively Coupled Plasma Mass Spectrometry (ICP-MS). EFA revealed distinct morphological differences: dorsal scales were broader anteriorly and more tapered posteriorly than anal scales. Microchemically, while Ca and Mg predominated in both types, significant differences were observed in Mn/Ca (*p* = 0.0007) and Mg/Ca (*p* = 0.017) ratios. Principal component analysis (PCA) suggested a trend of clear separation between the two scale regions based on their elemental signatures. Notably, dorsal scales exhibited higher elemental consistency and lower intra-group variability. These results suggest that dorsal scales may be more suitable for standardized sampling. Prioritizing the dorsal region will minimize sampling bias and enhance the reliability of non-lethal microchemical studies in fisheries research.

## Simple Summary

The Yangtze River is home to many unique fish species, but dam construction and overfishing have pushed several, such as *Coreius guichenoti*, to the brink of extinction. To protect these species non-destructively, scientists can utilize fish scales—a renewable and easily collected biological tissue—to reconstruct their life history and environmental exposure. Crucially, before scales can be widely applied in monitoring, it must be determined whether scales from different body regions record consistent biological information. In this study, we compared scales collected from the dorsal and anal regions of *C. guichenoti*. Our results show that these scales differ significantly in both morphology and chemical composition, indicating that regional differences exist between scale types, which may influence their interchangeability in specific analytical contexts. This finding highlights the necessity of standardized sampling from consistent body locations in fish research. Ultimately, our study helps establish a reliable, non-lethal methodology to better understand and conserve this endangered species.

## 1. Introduction

*Coreius guichenoti* (Sauvage & Dabry de Thiersant, 1874), belonging to the Cyprinidae family, Gobioninae subfamily, and *Coreius* genus [1], is an economically and ecologically important species endemic to the upper Yangtze River [2,3]. Its life history is characterized by long-distance, stage-specific potamodromous migrations [4,5]. In recent years, however, hydropower development and habitat degradation have led to a sharp population decline, and the species is now listed as a State Second-Class Protected Animal in China [6]. In this context, the development of non-lethal techniques to obtain critical information on its life history and habitat use is urgently needed to inform conservation strategies.

Fish scales, as a renewable and non-lethal sampling material, can continuously archive physiological and environmental information throughout the life of the fish, offering considerable potential for reconstructing life histories. This potential is evident in two main areas. Morphologically, scale characteristics have been proven to be reliable taxonomic tools, having been successfully used to differentiate populations of algivorous cyprinid fishes in Iran and to distinguish 11 fish species from New Zealand and Turkey [7,8]. In terms of microchemistry, the trace element composition of scales (e.g., Sr/Ca and Ba/Ca ratios) reflects the chemical fingerprints of the ambient water, and has been widely used as a tool for tracing life histories and migratory patterns. Specific applications include distinguishing between marine and freshwater habitat origins, discriminating wild from farmed populations, and reconstructing the salinity migration history of estuarine fishes [9,10,11].

However, the reliability of scales as carriers of environmental information is constrained by two key factors. First, the deposition of trace elements is not a passive recording process; rather, it is subject to complex regulation by absorption–excretion dynamics, environmental parameters (e.g., water temperature, salinity), and physiological metabolism [12,13], which introduces uncertainty into the interpretation of environmental signals. Second, the developmental biology of scales themselves—particularly potential differences in the timing of formation and growth rates among scales from different body regions—may further affect the homogeneity and comparability of the information recorded. In *C. guichenoti*, scales first appear in the pre-lateral line region at 22.2 days post-hatching and then progressively extend posteriorly [14], it is hypothesized that scales from dorsal and anal regions may possess non-equivalent growth histories. However, whether these region-specific differences are systematic and consistent remains unclear.

Notably, previous studies on scale morphology and microchemistry have employed scales from different body regions, including dorsal, lateral-line, and anal regions, depending on research objectives and species characteristics [15,16,17]. However, a standardized sampling location has not been established, and the comparability of results derived from different regions remains largely unverified. This raises a critical methodological question: whether scales from different body regions can be used interchangeably in morphological and microchemical analyses.

Therefore, before scales can be applied to reconstruct the life history of endangered species such as *C. guichenoti*, it is essential to clarify whether scales from different body regions exhibit systematic differences in morphological structure and trace element deposition. Using *C. guichenoti* as a model species, this study aims to systematically compare the morphology and key trace element distribution patterns of scales from dorsal and anal regions. These two regions were selected to represent contrasting functional and environmental conditions rather than arbitrary sampling. The objective of this study is to evaluate regional variability in scale characteristics and to provide empirical evidence for the standardization of sampling location, thereby supporting the application of non-lethal approaches in life history reconstruction.

## 2. Materials and Methods

### 2.1. Study Area and Sampling

**Figure 1:**
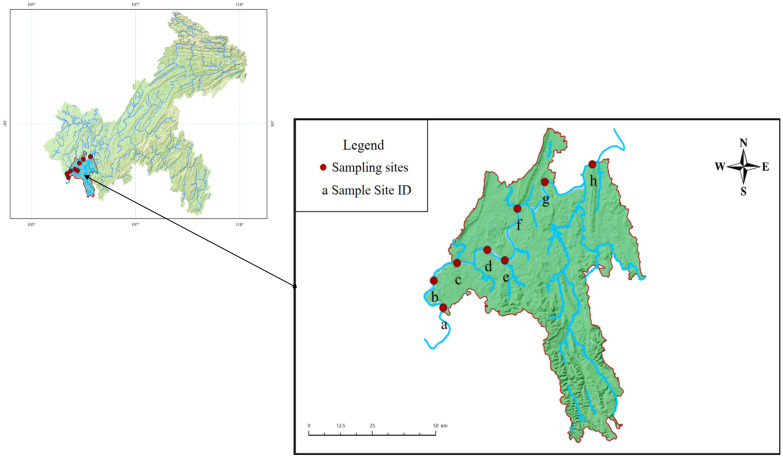
Rare and Endemic Fish National Nature Reserve in Chongqing of Yangtze River.

The sampling sites were located in the Chongqing section of the Yangtze River Reserve, extending from Yangshi Street in Shima Town to Diwei Bridge in Luohuang Town, Jiangjin District (Figure 1). The geographical coordinates of the study area range from 105°53′21″ E to 106°24′16″ E and from 28°55′35″ N to 29°20′34″ N. Samples were collected at monitoring stations established in nine towns—Shima, Zhutuo, Songgai, Zhuyang, Shimen, Baisha, Youxi, Degan, and Luohuang—during two periods: June to October 2014 and March to June 2015.

For this study, a total of 22 specimens of *C. guichenoti* were collected from the Jiangjin section of the Upper Yangtze River Rare Fish Reserve in Chongqing using gillnets and trap nets After capture, fish were held in aerated river water, measured for body length (to 0.1 cm) and weight (to 0.1 g), and scales were removed from the dorsal and anal regions. All fish were then released unharmed at the capture site. The average body length was 22.66 ± 2.18 cm, and the average body weight was 187.97 ± 81.08 g. After scale removal, the samples were transported to the laboratory in iceboxes and stored at −20 °C until further analysis.

### 2.2. Scale Morphological Preprocessing

**Figure 2:**
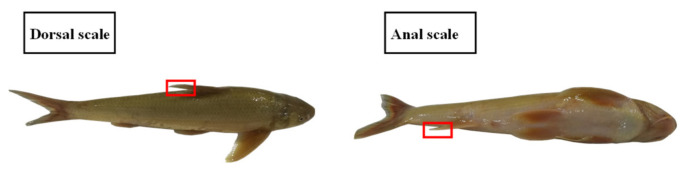
Positions of dorsal and anal scales collected from *C. guichenoti*.

**Figure 3:**
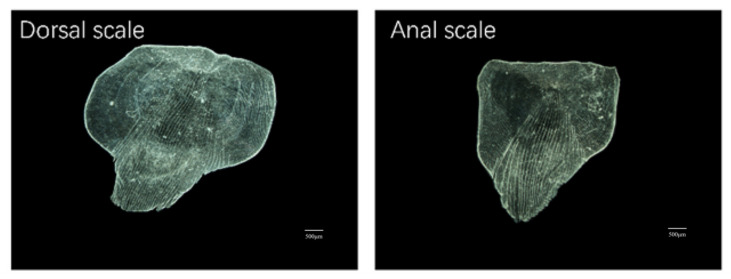
Photographs of dorsal and anal scales of *C. guichenoti*.

From each specimen, one dorsal scale (from the left side of the dorsal fin) and one anal scale (from the anal fin region) were collected (Figure 2, sampling positions are indicated). The scales were treated with a 5% KOH solution for 3–5 h with pure water for 3–5 h to remove organic tissues and surface contaminants prior to morphological and microchemical analyses, rinsed thoroughly with distilled water, pressed flat between two clean glass slides, and air-dried at room temperature for 48 h. Clear images of the prepared scales were then captured using a stereomicroscope (Motic SM171 microscope equipped with a Moticam S6 digital camera; Motic, Xiamen, China) (Figure 3).

### 2.3. Scale Morphological Analysis

**Figure 4:**
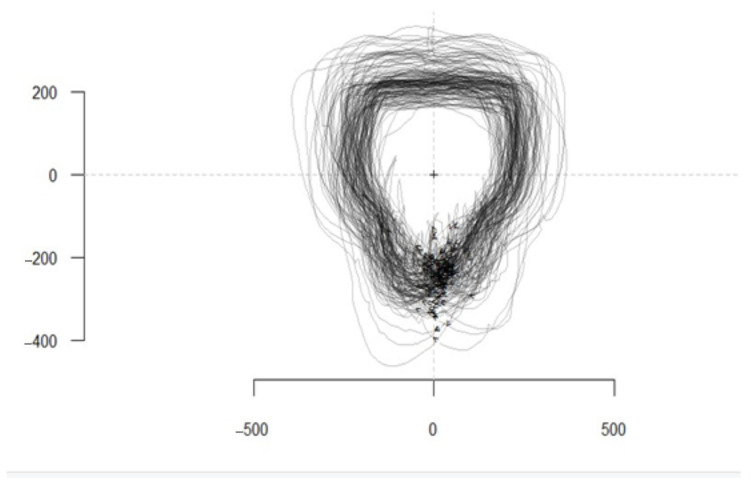
The result of the overlapping of all *C. guichenoti* scales.

This study employed elliptic Fourier analysis (EFA) to compare shape differences between dorsal and anal scales. Using the “Momocs” R package, raw images were automatically processed to extract scale outlines, and the extracted contours were checked to ensure closure and consistent point ordering, with no manual correction applied to avoid subjective bias, followed by standardization procedures including alignment of starting points, coordinate centralization, and isometric scaling to eliminate size effects (Figure 4). Each scale outline was converted into a series of (x, y) coordinates and subjected to elliptic Fourier transformation. To balance the capture of essential shape information against model complexity, the optimal number of harmonics was determined based on their cumulative contribution to shape variance. The first 15 harmonic coefficients (each containing four Fourier descriptors: An, Bn, Cn, and Dn) were retained, as they collectively accounted for ≥99% of the cumulative shape variation. This threshold ensures that biologically significant shape variation is adequately represented while effectively filtering out high-frequency components likely attributable to measurement noise. Finally, the average outline shapes for each scale group were computed, with all analyses performed in R v4.2.0.

### 2.4. Scale Microchemical Preprocessing

From the 22 specimens, 10 individuals with intact scales were selected based for ICP-MS analysis on the availability of suitable scales. The scales were cleaned in an ultrasonic (YM-008S, China) cleaner for 10–20 min to remove surface contaminants, dried, and then subjected to acid digestion prior to analysis.

### 2.5. Scale Microchemistry Analysis

This study analyzed trace elements (Mg, Ca, Sr, Mn, and Ba) in anal and dorsal scales using inductively coupled plasma mass spectrometry (ICP-MS; PerkinElmer NexION 1000G; China) following acid digestion and analysis of solution-based samples. All samples were fully digested into aqueous solutions prior to analysis, and only particle-free solutions were introduced into the instrument. The instrument was operated under the following conditions: radio frequency (RF) power 1600 W, plasma gas flow 15 L/min, auxiliary gas flow 1.2 L/min, nebulizer gas flow 1.0 L/min, and dwell time 50 ms per isotope. Data were acquired in peak hopping mode with three replicates per sample. During data preprocessing, the normality of Me/Ca data was first assessed using the Shapiro–Wilk test. For element ratios that did not follow a normal distribution, powerTransform and BoxCox transformations were applied to meet the requirements of multivariate normality. Subsequently, independent samples *t*-tests were performed on the element ratios, followed by principal component analysis (PCA). All elemental measurements were successfully obtained for both dorsal and anal scales from the 10 individuals, and no missing values were detected.

Potential multivariate outliers were assessed using Mahalanobis distance based on the element-to-calcium ratio dataset.

### 2.6. Statistical Analyses

All statistical analyses were performed in R (version 4.2.0) using packages including ggplot2 (3.4.0), dplyr (1.1.0), and tidyr (1.3.0). Normality was assessed using the Shapiro–Wilk test, and appropriate transformations were applied when necessary. Differences between dorsal and anal scales were evaluated using independent samples *t*-tests, with effect sizes (Cohen’s d) calculated to quantify the magnitude of differences. Principal component analysis (PCA) was conducted to assess multivariate variation in elemental ratios. Figures were generated using ggplot2, and Excel 2019 was used for preliminary data organization and management.

## 3. Results

### 3.1. Scale Morphology

**Figure 5:**
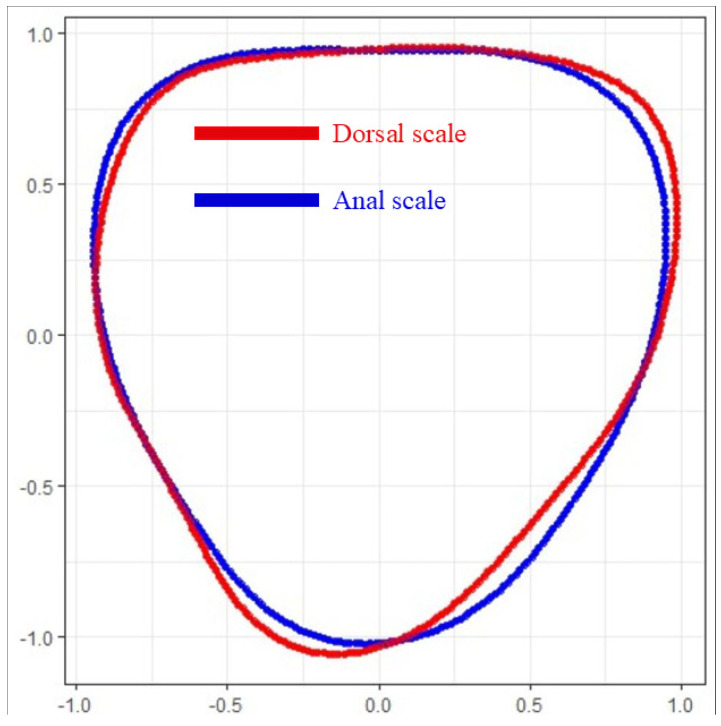
Average shapes reconstructions of anal-dorsal scales through elliptic Fourier analysis.

The average shapes reconstructed by elliptic Fourier analysis (EFA) are shown in Figure 5. The results reveal that anal and dorsal scales share overall morphological similarity, but exhibit subtle variations: the dorsal scales display a broader basal region, and their posterior section is characteristically tapered and elongated; in contrast, the anal scales have a relatively narrower basal region and a more rounded posterior profile, with dorsal scales being slightly larger overall. The mean area of dorsal scales (3.24) was slightly larger than that of anal scales (3.16), supporting this tendency.

### 3.2. Scale Microchemistry

**Figure 6:**
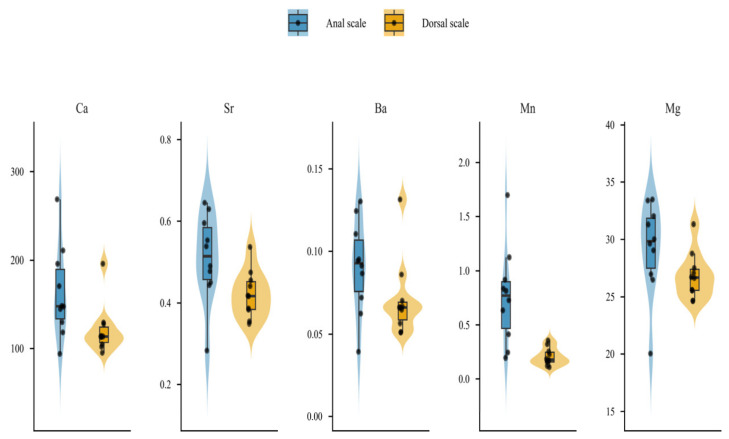
Concentrations of Sr, Ba, Mn, Mg, and Ca in Anal and Dorsal Scales.

The analysis of dorsal and anal scales of *C. guichenoti* focused on five elements: Sr, Ba, Mn, Mg, and Ca, all of which were detected above their respective detection limits. Among the measured elements, calcium was the most abundant (dorsal scales: 121.09 ± 28.4 μmol/L; anal scales: 162.96 ± 50.86 μmol/L), followed by magnesium (dorsal scales: 26.85 ± 2.04 μmol/L; anal scales: 29.25 ± 4.02 μmol/L). The concentrations of strontium (dorsal: 0.42 ± 0.06 μmol/L; anal: 0.51 ± 0.11 μmol/L), manganese (dorsal: 0.21 ± 0.08 μmol/L; anal: 0.76 ± 0.44 μmol/L), and barium (dorsal: 0.07 ± 0.02 μmol/L; anal: 0.09 ± 0.03 μmol/L) were substantially lower. Consequently, calcium was used as the internal standard for normalization in subsequent analyses (Figure 6).

**Figure 7:**
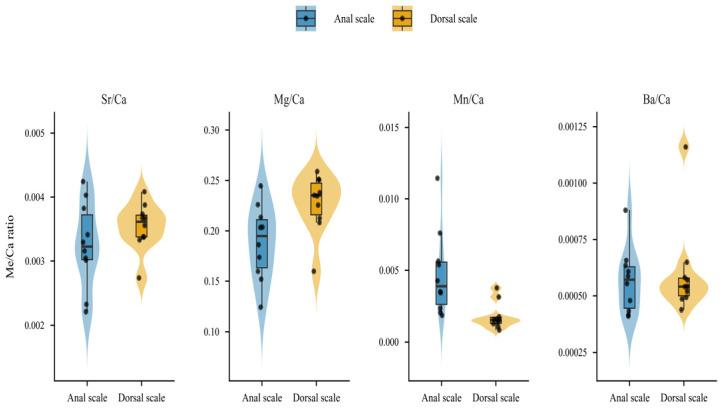
Distribution of Sr/Ca, Ba/Ca, Mn/Ca, Mg/Ca in Anal and Dorsal Scales.

Analysis of the dispersion of element-to-calcium ratios in dorsal and anal scales revealed distinct patterns: the ratios were more clustered in dorsal scales, whereas they exhibited greater dispersion in anal scales. Specifically, the Ba/Ca ratio was low and narrowly distributed in both scale types. The Mg/Ca ratio was higher in dorsal scales than in anal scales, while the Mn/Ca ratio showed the opposite trend. The distribution pattern of Sr/Ca resembled that of Ba/Ca, but at a higher concentration level. Shapiro–Wilk tests indicated that, among the metal-to-calcium ratio datasets, only the Sr/Ca and Mg/Ca ratios for both scale types conformed to a normal distribution. To meet the assumptions of parametric testing, power transformations were applied: y^−1.8^ for Ba/Ca and y^−0.34^ for Mn/Ca. Independent samples *t*-tests revealed no significant differences between anal and dorsal scales for either the Sr/Ca (*p* = 0.25, Cohen’s d = 0.53) or Ba/Ca ratios (*p* = 0.65, d = 0.18). In contrast, the Mn/Ca ratio differed highly significantly between the two scale (*p* = 0.0007, Cohen’s d = −1.35), indicating a large effect size with higher values in anal scales. Similarly, the Mg/Ca ratio also showed a significant difference (*p* = 0.017, Cohen’s d =1.17), also representing a large effect size, with higher values observed in dorsal scales (Table 1, Figure 7).

**Figure 8:**
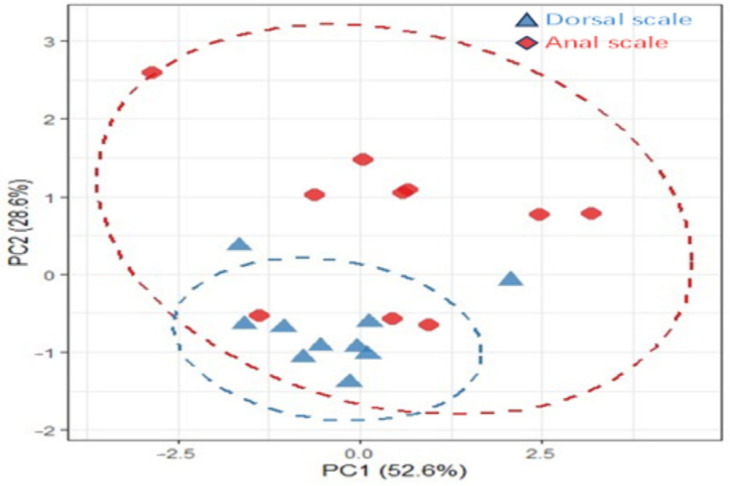
PCA of Trace Elements in Anal and Dorsal Scales.

**Figure 9:**
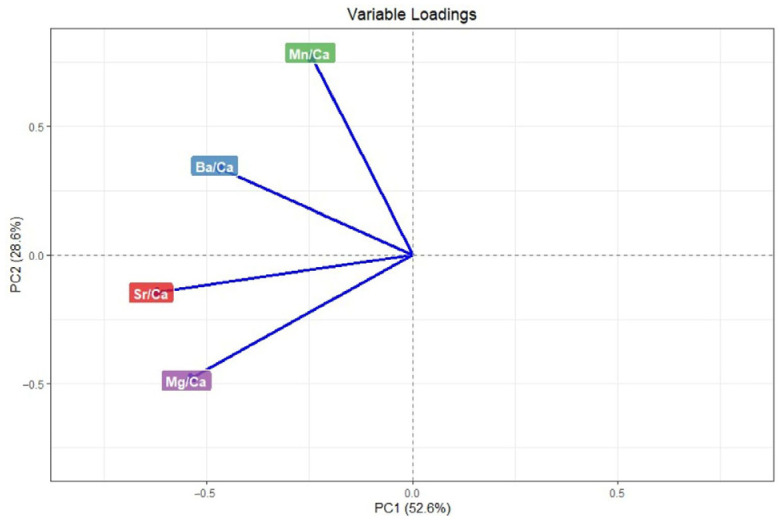
Variable loading plot of elemental-to-calcium Me/Ca in PCA.

Principal component analysis (PCA) of scale Me/Ca ratios suggested a trend of differentiations in trace element signatures between the dorsal and anal scales of *C. guichenoti*. The first two principal components (PC1 and PC2) explained 52.6% and 28.6% of the total variance, respectively, cumulatively accounting for 81.2%. The score plot showed a weak separation trend without statistical support: dorsal scale samples formed a relatively tight cluster in the negative quadrants of both PC1 and PC2, suggesting greater homogeneity in elemental composition. In contrast, anal scale samples were primarily located in the positive region of PC2 and displayed considerable dispersion along PC1. Loadings analysis indicated thatPC1 was associated with opposing contributions from Sr/Ca and Mg/Ca (negative loadings) versus Ba/Ca (positive loading), implying a compositional gradient among these elements. PC2 was primarily driven by Mn/Ca, signifying element-specific enrichment pathways in the scales. The numerical PCA loading values corresponding to the loading plot are provided in Table 2. In summary, Mn/Ca and Mg/Ca contributed most to the observed variation along PC axes, while anal scales showed more pronounced enrichment of Mn/Ca and Ba/Ca, especially a higher contribution of Mn/Ca. Conversely, dorsal scales were characterized by greater accumulation of Sr/Ca and Mg/Ca, with the difference in Mg/Ca being particularly notable. Although one anal scale sample appeared visually distant from the main cluster in the PCA score plot (Figure 8 and Figure 9), outlier diagnostics based on Mahalanobis distance indicated that no samples exceeded the statistical threshold for multivariate outliers. Therefore, all samples were retained in the analysis.

However, PERMANOVA analysis indicated that this apparent separation was not statistically significant (F = 0.216, R^2^ = 0.012, *p* = 0.65), suggesting that the overall multivariate elemental composition did not differ significantly between dorsal and anal scales.

## 4. Discussion

### 4.1. Morphological Differential Characteristics Between Dorsal and Anal Scales

Scale morphology varies across different body regions [17,18]. These differences may be associated with environmental and biological factors; however, the present study does not directly test these mechanisms [19,20]. Hydrostatic pressure is an important environmental factor in aquatic systems [21]; however, its role in shaping scale morphology remains unclear and was not examined in this study. In this study, the dorsal scales of *C. guichenoti* were longer than the anal scales. Similar regional variation has been reported in *Sparus aurata*, where dorsal scales are larger than those in other body regions [22]. The observed shape differences may be related to functional adaptation to different body locations. The slender and sharply pointed morphology of the posterior region of the dorsal scale may be associated with hydrodynamic influences [23]. In contrast, the broader and more rounded anterior region of dorsal scales likely corresponds to the prominent postcephalic hump of the fish body. Moreover, age determination studies on *Ptychobarbus dipogon* [24] and *Cyprinion macrostomum* [25] have demonstrated that dorsal scales are more effective for age estimation. This feature may facilitate clearer annulus identification, as previously reported in other cyprinid species. Consequently, when conducting classification and age determination of *C. guichenoti* based on scale morphology, it is methodologically preferable to use comparative analyses of scales from same anatomical regions [26].

### 4.2. Microelement Differences Between Dorsal and Anal Scales

As a calcified structure [27], fish scales serve as an internal calcium reservoir and play a central regulatory role in maintaining calcium homeostasis [28]. Consistent with this physiological function, calcium content was significantly higher than other trace elements. Notably, magnesium levels were markedly greater than strontium, barium, and manganese, consistent with findings from Maros River carp scales [29]. Previous studies have demonstrated that magnesium concentrations remain relatively stable under varying environmental conditions [30], and research on red-spotted grouper has further confirmed that dietary calcium has limited influence on scale magnesium content [31]. Further investigations have revealed a complex relationship between magnesium deposition in calcified tissues and environmental magnesium concentrations, primarily governed by the intrinsic regulation of polyphosphate levels in mineralized tissues [32]. The specific absorption kinetics underlying this process require further elucidation. Regarding the distribution patterns of other elements, the higher strontium content relative to barium may be attributed to the greater propensity of hydroxyapatite crystals to incorporate Sr^2+^. Given its ionic radius more closely resembles that of Ca^2+^, Sr^2+^ substitutes more readily for calcium within the hydroxyapatite lattice compared to the larger Ba^2+^ ion. Similarly, manganese levels were higher than those of barium, which is consistent with previous findings [33].

This study investigated the benthic omnivorous fish *C. guichenoti* and found that the concentrations of all trace elements were higher in anal scales than in dorsal scales. This pattern may reflect localized environmental exposure, although this study did not directly measure habitat-specific variables. However, analyses of muscle tissues from fish occupying different ecological niches have revealed higher concentrations of Zn and Co in pelagic species compared to benthic fishes [34]. Furthermore, a comparative study of scale elements in the benthic fish *L. calcarifer* and other pelagic fishes demonstrated that *L. calcarifer* exhibited the lowest levels of Cu, Al, Mg, and Mn, in contrast to the findings of the present study [35]. These discrepancies may arise from a combination of environmental factors and species-specific characteristics. Previous research has indicated that mineral concentrations in teleost scales can vary within a range of 16% to 59% [36], and their elemental composition is closely associated with the hydrochemical conditions of their habitat. Changes in environmental parameters such as water temperature and salinity can influence scale element deposition by altering the solubility and bioavailability of elements in the water column. Scale mineralization is a continuous process throughout the life of fish, and both environmental and physiological factors may influence elemental accumulation [37]. These findings suggest that elemental differences between scale regions are influenced by multiple interacting factors rather than a single dominant mechanism.

To reduce the “noise” in trace element composition caused by differences in scale growth rates, we analyzed elemental ratios (Me/Ca) in both anal and dorsal scales to more clearly reflect environmental signals. The lower dispersion of elemental ratios in dorsal scales of *C. guichenoti* indicates a more consistent elemental deposition pattern among individuals, whereas anal scales exhibited greater variability. This may relate to the species’ demersal and omnivorous lifestyle: anal scales, being closer to the substrate, are more influenced by sedimentary environments. However, this study did not directly measure environmental variables, and this interpretation should be treated with caution. This variability may reflect localized physiological regulation or environmental heterogeneity.

Previous studies have shown that elemental ratios (Me/Ca) in calcified tissues such as scales are influenced by environmental factors including salinity and diet [38,39]. In this study, differences in Mg/Ca and Mn/Ca between dorsal and anal scales were significantly greater than those of Sr/Ca and Ba/Ca. This may be attributed to the deposition of magnesium and manganese being more strongly regulated by physiological processes than by environmental concentrations [40]. Magnesium and manganese are known to be involved in physiological processes, suggesting that their incorporation may be influenced by biological regulation [41,42]. Therefore, their distribution in scales may reflect biochemical demands more than external concentrations, and intra-scale variability may exceed inter-environmental differences [33]. This pattern may be influenced by a combination of physiological regulation and environmental exposure.

Principal component analysis revealed that Sr/Ca, Ba/Ca, Mn/Ca, and Mg/Ca ratios contributed to the observed variation between anal and dorsal scales in *C. guichenoti,* indicating a trend of differentiation, although the overall multivariate differences were not statistically significant based on PERMANOVA results. This suggests that while certain elemental ratios (Mn/Ca and Mg/Ca) contribute to variation between scale types, the overall separation remains limited. Both PCA and *t*-test results identified Mn/Ca and Mg/Ca as key indicators for differentiating the two scale types, with dorsal scales exhibiting significantly higher Mg/Ca ratios, while anal scales had significantly higher Mn/Ca ratios.

The distinct Mn/Ca and Mg/Ca signatures between anal and dorsal scales in this species may reflect complex mechanisms of environmental adaptation and physiological regulation. Existing studies demonstrate divergent relationships between temperature and Mg/Ca in calcified tissues: for instance, otolith Mg/Ca in *Argyrosomus japonicus* shows a positive correlation with environmental temperature [43], whereas several marine fish otolith studies found no such association with water temperature [44]. The elevated Mg/Ca ratios observed in dorsal scales in our study may correspond to their faster growth rates [45], which is consistent with their elongated morphology.

Regarding Mn/Ca, although Japanese flounder otolith studies indicated no significant correlation with salinity or temperature [13], European flounder *Platichthys flesus* under hypoxic conditions demonstrated significantly increased Mn/Ca ratios [46]. As an essential element in calcification processes, Mn/Ca is typically constrained by mineralization rates, with slower-growing calcified tissues generally displaying lower Mn/Ca ratios [47]. This interpretation is tentative, as direct measurements of scale growth rates were not conducted. This discrepancy highlights the complexity of trace element incorporation in scales and suggests that existing models may not fully apply to this species.

This study has several limitations. Environmental variables such as water chemistry and sediment composition were not directly measured, limiting the ability to link elemental composition to external conditions. In addition, the sample size was relatively limited, and only two scale regions were analyzed. Future studies incorporating controlled experiments and comparisons with other calcified tissues are needed. Therefore, interpretations regarding environmental and physiological mechanisms should be considered tentative. Despite these limitations, this study provides important implications for non-lethal ecological research. The observed differences between dorsal and anal scales indicate that sampling location should be standardized. Dorsal scales, with lower variability, may be more suitable for age determination and environmental reconstruction in *C. guichenoti*.

## 5. Conclusions

This study conducted a systematic comparative analysis of dorsal and anal scales in *C. guichenoti* through the integrated application of elliptic Fourier analysis (EFA) and inductively coupled plasma mass spectrometry (ICP-MS). Morphological observations revealed that dorsal scales exhibit an elongated morphology characterized by a broad, rounded anterior and a slender, pointed posterior, suggesting potential differences in functional adaptation associated with their body positions. At the microchemical level, although both scale types were rich in calcium and magnesium, they displayed significantly distinct elemental distribution patterns: dorsal scales showed relatively stable trace element compositions, whereas anal scales exhibited greater variability. Notably, the magnesium-to-calcium (Mg/Ca) and manganese-to-calcium (Mn/Ca) ratios demonstrated clear regional specificity—dorsal scales had significantly higher Mg/Ca ratios, while anal scales showed significantly higher Mn/Ca ratios. These two elemental ratios thus may serve as useful indicators for distinguishing between dorsal and anal scales, although further validation is needed. Collectively, these findings reveal pronounced regional heterogeneity in the scales of *C. guichenoti* at both morphological and microchemical levels, suggesting that scale development may be influenced by localized physiological processes and environmental conditions. Given the relatively stable trace element composition in dorsal scales, they are relatively more suitable than anal scales for use as standardized sampling material in life history reconstruction and habitat identification studies. From a methodological perspective, we recommend that future scale-based studies on life history tracking and habitat reconstruction adopt standardized sampling protocols from consistent body locations to enhance data comparability and reproducibility, thereby strengthening the reliability of research conclusions.

## Figures and Tables

**Table 1:** Results of *t*-tests for Me/Ca between anal and dorsal scales.

Element Ratio	Levene’s Test *p*-Value	*t*-Test *p*-Value	Cohen’s d
Sr/Ca	0.13	0.25	0.52
Ba/Ca	0.23	0.65	0.17
Mn/Ca	0.78	0.0007	−1.35
Mg/Ca	0.30	0.02	1.17

**Table 2:** PCA loadings of element-to-calcium ratios (Me/Ca) in dorsal and anal scales of *C. guichenoti*.

Element Ratio	PC1	PC2
Sr/Ca	−0.0628	−0.053
Ba/Ca	0.551	−0.277
Mn/Ca	0.114	−0.869
Mg/Ca	−0.537	−0.407

## Data Availability

The data that support the findings of this study are available within the article.
